# The MK2 cascade mediates transient alteration in mGluR‐LTD and spatial learning in a murine model of Alzheimer's disease

**DOI:** 10.1111/acel.13717

**Published:** 2022-09-22

**Authors:** Lucia Privitera, Ellen L. Hogg, Marcia Lopes, Luana B. Domingos, Matthias Gaestel, Jürgen Müller, Mark J. Wall, Sonia A. L. Corrêa

**Affiliations:** ^1^ Bradford School of Pharmacy and Medical Sciences University of Bradford Bradford UK; ^2^ School of Medicine, Ninewells Hospital University of Dundee Dundee UK; ^3^ Barts and the London School of Medicine Queen Mary University of London Malta Campus Victoria Malta; ^4^ Institute of Cell Biochemistry Hannover Medical University Hannover Germany; ^5^ School of Life Sciences University of Warwick Coventry UK; ^6^ Faculty of Science and Engineering Manchester Metropolitan University Manchester UK

**Keywords:** APP/PS1 mouse, Arc/Arg3.1, hippocampus, mGluR5 signalling, p38 MAPK signalling, synaptic plasticity

## Abstract

A key aim of Alzheimer disease research is to develop efficient therapies to prevent and/or delay the irreversible progression of cognitive impairments. Early deficits in long‐term potentiation (LTP) are associated with the accumulation of amyloid beta in rodent models of the disease; however, less is known about how mGluR‐mediated long‐term depression (mGluR‐LTD) is affected. In this study, we have found that mGluR‐LTD is enhanced in the APP_swe_/PS1dE9 mouse at 7 but returns to wild‐type levels at 13 months of age. This transient over‐activation of mGluR signalling is coupled with impaired LTP and shifts the dynamic range of synapses towards depression. These alterations in synaptic plasticity are associated with an inability to utilize cues in a spatial learning task. The transient dysregulation of plasticity can be prevented by genetic deletion of the MAP kinase‐activated protein kinase 2 (MK2), a substrate of p38 MAPK, demonstrating that manipulating the mGluR‐p38 MAPK‐MK2 cascade at 7 months can prevent the shift in synapse dynamic range. Our work reveals the MK2 cascade as a potential pharmacological target to correct the over‐activation of mGluR signalling.

## Graphical abstract

Disruption of synapse potentiation is associated with cognitive impairment in Alzheimer's disease (AD), yet the mechanisms mediating this dysfunction remain unknown. We showed that over‐activation of mGluR‐LTD shifts the synaptic capacity range towards depression and genetic deletion of MK2 in AD corrects over‐activation of mGluR‐LTD and restores the depressed synaptic range to normal level.

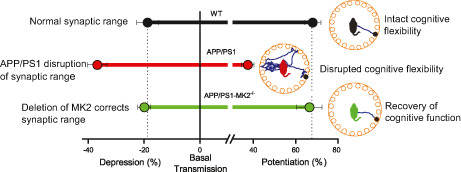

## 1 INTRODUCTION

Alzheimer's disease (AD) is an irreversible, progressive brain disorder whose neuropathological hallmark includes neuronal death leading to memory loss and cognitive decline (Li & Selkoe, 2020). Early cognitive changes are believed to be, at least in part, due to dysfunction in communication between excitatory glutamatergic neurons (Wang & Reddy, 2017). In the hippocampus, two complementary forms of glutamatergic synaptic plasticity, long‐term potentiation (LTP) and long‐term depression (LTD) are believed to act dynamically to mediate storage of spatial information (Nabavi et al., 2014; Takeuchi et al., 2014). Two mechanistically distinct forms of LTD coexist at the CA3‐CA1 hippocampal synapses: one is induced by activation of N‐methyl‐D‐aspartate receptors, NMDAR‐LTD and the other is induced by the activation of group I metabotropic glutamate receptors, mGluR‐dependent LTD (Gladding et al., 2009; Reiner & Levitz, 2018).

Numerous studies have demonstrated that LTP is impaired in the hippocampus of the APP_swe_/PS1dE9 (APP/PS1) mouse model of AD and the weakening in synaptic strength correlates with a decline in spatial working memory (Heneka et al., 2013; Ma et al., 2013). However, how mGluR signalling regulates this process is not well characterized. Supporting the idea that mGluR‐LTD may be affected at early stages of the disease are findings showing that accumulation of amyloid beta (Aβ) oligomers is associated with excessive activation of mGluR5 signalling and that pharmacological antagonism of mGluR5 ameliorates cognitive impairments in the APP/PS1 mouse (Hamilton et al., 2016; Renner et al., 2010; Um et al., 2013). Aβ oligomers have been shown to bind to mGluR5 which reduces their plasma membrane mobility and leads to receptor accumulation at the cell surface (Renner et al., 2010). As the amount of Aβ increases with age in the APP/PS1 mouse, it is expected that over‐activation of mGluR5 signalling contributes to the impaired synaptic plasticity. The p38 MAPK cascade is a strong candidate to mediate these early changes. Previous studies have shown that Aβ‐mediated inhibition of LTP is mediated by activation of mGluR5 and p38 (Wang et al., 2004). Furthermore, inhibition of p38 blocks mGluR‐LTD at the CA3‐CA1 synapses (Bolshakov et al., 2000; Moult et al., 2008) suggesting that manipulation of p38 activity may rescue Aβ‐mediated impairments in LTP. Supporting this idea, we demonstrated that knockdown of MAPK‐activated protein kinase 2 (MK2) protein, a substrate of p38, in rodents markedly reduces mGluR‐LTD amplitude, with no effect on either LTP or NMDAR‐mediated LTD at CA3‐CA1 synapses (Correa & Eales, 2012; Privitera et al., 2019). An interesting finding from this study is that although LTP was intact in the MK2 knockout (KO) mouse, the mGluR‐mediated priming of LTP amplitudes was impaired, indicating a functional role for the mGluR5‐p38‐MK2 cascade regulating LTP (Privitera et al., 2019). Consistent with intact LTP, the MK2 KO mouse showed no deficits in learning a spatial task, while reversal learning was impaired, consistent with impaired mGluR‐LTD (Privitera et al., 2019). Although the role of the MK2 cascade mediating cellular responses to stress and inflammatory signals, including synthesis and release of pro‐inflammatory factors in immune cells, is well‐characterized (Menon & Gaestel, 2018; Soni et al., 2019), the mechanism by which MK2 signalling mediates neuronal response to inflammatory factors and neurotransmitter is poorly understood. Together, these findings suggest that MK2 may mediate the over‐activation of mGluR5 signalling seen in the APP/PS1 mouse. To address this, we deleted MK2 in APP/PS1 mice and found that enhanced mGluR‐LTD is a transient event present at 7 months but returned to wild‐type levels at 13 months of age and that genetic deletion of MK2 in the APP/PS1 mouse is sufficient to prevent the alterations in synaptic plasticity observed at 7 months.

## 2 RESULTS

### 2.1 Deletion of MK2 in APP/PS1 mice prevents alterations in synaptic plasticity at 7 months

Over‐activation of mGluR5 is reported to be associated with cognitive impairment in APP/PS1 male mice (Hamilton et al., 2016), and excessive mGluR5 signalling activation is restricted to early stages of the disease (Abd‐Elrahman et al., 2020). There is evidence showing that Aβ‐mediated over‐activation of mGluR signalling requires the activation of p38 (Chen et al., 2013) and activation of the p38 cascade is required for mGluR‐LTD (Bolshakov et al., 2000; Moult et al., 2008). Here, we hypothesized that the p38‐MK2 signalling pathway mediates the mGluR5 over‐activation observed in APP/PS1 mice. To address this, we generated a novel genetically modified mouse by crossing APP/PS1 with MK2^−/−^ mice. APP/PS1xMK2^−/−^ mice were born with expected Mendelian ratios, with no differences in mortality rate or in weight of heterozygotes APP/PS1XMK2^+/−^ or homozygotes APP/PS1XMK2^−/−^ when compared with MK2^+/+^ (WT) and APP/PS1^−/−^ (WT) mice (Figure S1A–D). Deletion of MK2 in APP/PS1 (APP/PS1XMK2^−/−^) mice was confirmed by PCR analysis (Figure S1E).

**FIGURE 1:**
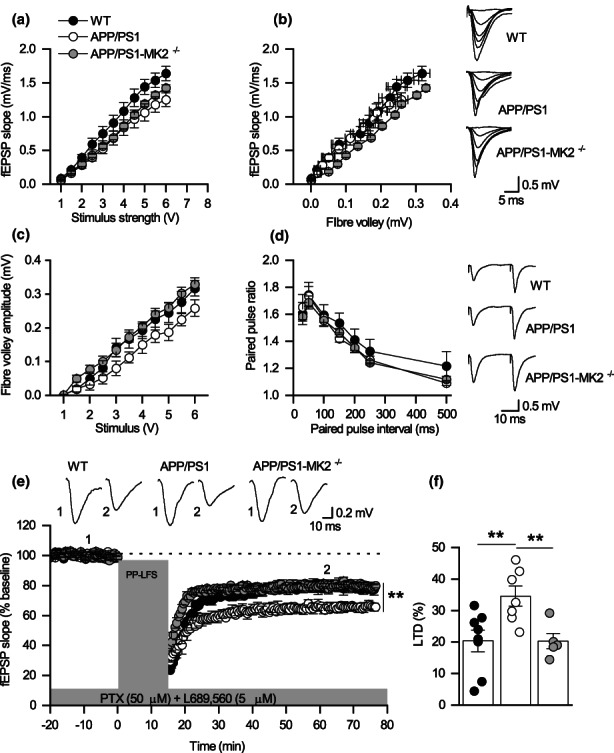
Deletion of MK2 in APP/PS1 mice prevents the enhancement of mGluR‐LTD observed at 7 months. (a) Mean fEPSP slope against stimulus strength for WT (*n* = 9), APP/PS1 (*n* = 7) and APP/PS1‐MK2^−/−^ (*n* = 5). No significant difference was observed between the three genotypes (*p* = 0.101). (b) Mean fEPSP slope against mean fibre volley amplitude WT (*n* = 5), APP/PS1 (*n* = 6) and APP/PS1‐MK2^−/−^ (*n* = 5). Inset: Superimposed fEPSPs at different stimulus strengths (1–6 V) from WT, APP/PS1 and APP/PS‐MK2^−/−^ mice. (c) Mean volley amplitude against stimulus strength (V). (d) Mean PPR against paired‐pulse interval for WT (*n* = 9), APP/PS1 (*n* = 9) and APP/PS1‐MK2^−/−^ (*n* = 5, *p* = 0.618). Inset, representative traces at interval of 50 ms from WT, APP/PS1 and APP/PS1‐MK2^−/−^ mice. (e) Normalized mean fEPSP slope against time WT (*n* = 8), APP/PS1 (*n* = 7) and MK2^−/−^‐APP/PS1 (*n* = 5) mice. After a 20‐min baseline (fEPSPs were stimulated at 40% of the maximum response), PP‐LFS used to induce LTD (WT: 79.6463 ± 3.4; APP: 65.43 ± 3.18; MK2^−/−^‐APP/PS1: 79.72 ± 2.44). There is a significant difference between genotypes (**p = 0.0076). Although LTD in APP/PS1‐MK2^−/−^ is not significant different from WT (*p* > 0.99), there is a significant difference between APP/PS1‐MK2^−/−^ and APP/PS1 (**p = 0.0029). Traces taken between 10–15 min (1) and LTD at 40–50 min (2) after LTD induction. (f) Mean percentage changes in LTD amplitudes between genotypes (WT: 20.35 ± 3.40; APP: 34.57 ± 3.18; MK2‐APP/PS1: 20.28 ± 2.44). Comparisons were carried out using one‐way ANOVA followed by Bonferroni's test. *p < 0.05, **p < 0.01.

To assess changes in synaptic plasticity, we measured LTP and mGluR‐LTD in interleaved hippocampal slices obtained from WT, APP/PS1 and APP/PS1XMK2^−/−^ mice at 7 months. Field excitatory postsynaptic potentials (fEPSPs) were recorded at the Schaffer collateral (SC)‐CA1 synapses. In agreement with a previous report (Gelman et al., 2018), there was no significant difference in the input/output (I/O) relationship (*p* = 0.101; Figure 1a), in the ratio of fEPSP slope to fibre volley amplitude (*p* = 0.078; Figure 1b,c) or in paired‐pulse facilitation (*p* = 0.618; Figure 1d) across genotypes. These findings suggest that synaptic transmission is not altered at presynaptic level. To further investigate whether there were postsynaptic changes in receptor density we recorded AMPAR‐mediated miniature excitatory postsynaptic currents (mEPSCs) in CA1 pyramidal cells obtained from the three genotypes. Although there were no significant differences in mEPSC amplitude across genotypes, there was a significant increase in mEPSC interval in APP/PS1 mice (Figure S2A–C). Deletion of MK2 in APP/PS1 mice prevented this increase in mEPSC interval (Figure S2D,E). Consistent with previous studies (Gelman et al., 2018; Heneka et al., 2013; Ma et al., 2013), LTP was significantly reduced in APP/PS1 mice (Figure S2F,G). Interestingly, the impairments in LTP observed in APP/PS1 mice were significantly reduced in APP/PS1XMK2^−/−^ mice (Figure S2F,G). In contrast, mGluR‐LTD was significantly enhanced in APP/PS1 mice (Figure 1e,f). Deletion of MK2 in APP/PS1 (APP/PS1XMK2^−/−^) mice returned LTD magnitude to similar levels to that recorded in WT mice (Figure 1e,f). This finding was unexpected as mGluR‐LTD is abolished in MK2 KO mice at 7 months (Privitera et al., 2019). The observation that the amount of mGluR‐LTD is only partially blocked in APP/PS1XMK2^−/−^ mice indicates that the pathogenic LTD seen in APP/PS1 mice must be mediated by different pathway(s).

To assess whether acute inhibition of MK2 had a similar effect as the constitutive deletion of the MK2 gene, hippocampal slices obtained from WT and APP/PS1 littermates at 7 months were perfused with the specific MK2 inhibitor PF 3644022 (3 μM) before the induction of LTD. Inhibition of MK2 blocked the expression of mGluR‐LTD at CA1‐CA3 hippocampal synapses in both genotypes (*p* = 0.523; Figure S3). One explanation for the partial inhibition of LTD in APP/PS1XMK2^−/−^ is that PP‐LFS mediated LTD recorded in slices from APP/PS1 mice may be mediated not only by mGluR5, but other pathways are involved. To assess this, hippocampal slices from WT and APP/PS1 mice were perfused with the mGluR5 inhibitor MTEP (1 μM) before PP‐LFS was used to induce LTD (Moult et al., 2008). Exposure to the mGluR5 inhibitor completely blocked mGluR‐LTD at SC‐CA1 synapses in both genotypes (*p* = 0.218; Figure S4), confirming that over‐activation of mGluR5 signalling mediates the enhanced mGluR‐LTD observed in APP/PS1 mice at 7 months. So why is LTD not abolished completely in APP/PS1XMK2^−/−^ mice? One possible explanation is a compensatory mechanism, possibly by a sister MAPK. Together, these findings suggest that the MK2 cascade enhances mGluR‐LTD associated with AD‐like neuropathology seen at 7 months.

### 2.2 Changes in Arc expression may underlie changes in mGluR‐LTD observed in APP/PS1 and APP/PS1XMK2 ^−/−^ mice

**FIGURE 2:**
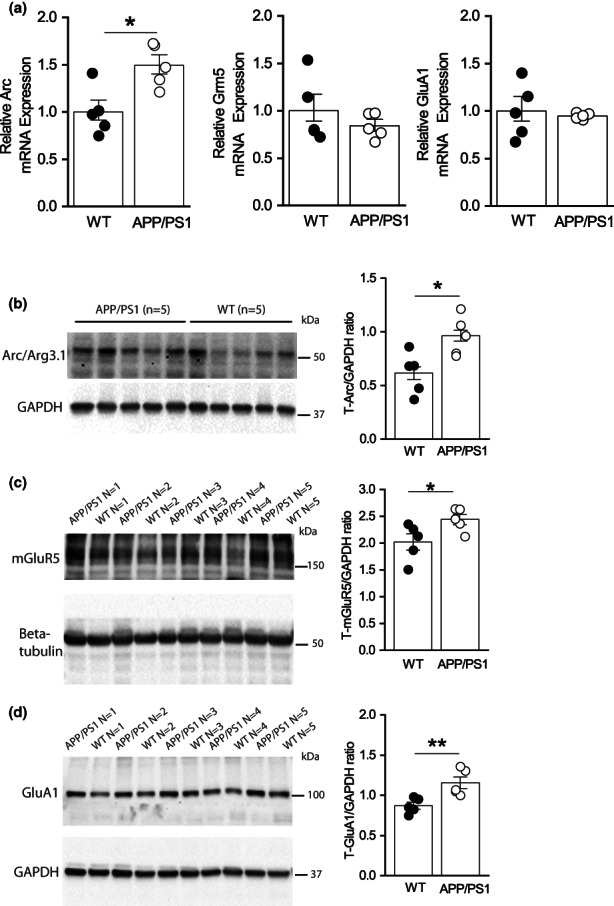
Arc mRNA and protein levels are increased in the hippocampus of APP/PS1 mice at 7 months of age. (a) Bar graphs showing mRNA levels of Arc (*p = 0.011), mGluR5 (*p* = 0.394) and GluA1 (*p* = 0.886) in the hippocampus of APP/PS1 compared to WT mice (*n* = 5 mice for each genotype). (b–d) Blots and bar graphs showing protein expression levels of Arc (*p = 0.019); mGluR5 (*p = 0.0493) and GluA1 (**p = 0.0095) in APP/PS1 compared to WT mice (*n* = 5 mice for each genotype). Unpaired Student's *t*‐test was used to compare mRNA and protein. *p ≤ 0.05, **p ≤ 0.01 and ***p ≤ 0.0001.

**FIGURE 3:**
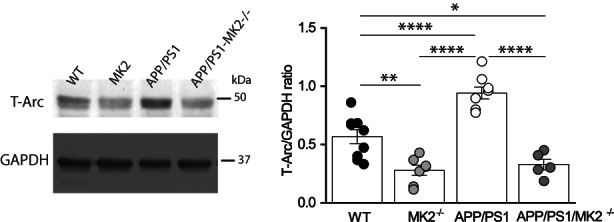
Deletion of MK2 normalizes Arc protein expression levels are in APP/PS1 mice at 7 months. Blots showing Arc protein levels in hippocampal lysate of WT (*n* = 9), MK2^−/−^ (*n* = 7) APP/PS1 (*n* = 8) and APP/PS1‐MK2^−/−^ (*n* = 5) mice at 7 months of age. The significant increase in Arc protein seen in lysates from APP/PS1 mice is reduced in the lysates from APP/PS1‐MK2^−/−^ mice (WT vs. MK2 KO, **p = 0.0018; APP/PS1 vs. MK2^−/−^, ****p < 0.0001; APP/PS1 vs. WT, ****p < 0.0001; WT vs. APP/PS1 MK2^−/−^, *p = 0.0140 and APP/PS1 vs. APP/PS1‐MK2^−/−^, ****p < 0.0001). One‐way ANOVA followed by holm–Sidak test. *p ≤ 0.05, **p ≤ 0.01 and ****p ≤ 0.0001.

As mGluR‐LTD was enhanced in APP/PS1 mice, we asked whether expression of key proteins mediating mGluR‐LTD was altered in the hippocampus of APP/PS1 mice at 7 months. To address this, we used a half‐brain from WT and APP/PS1 at 7 months to extract RNA from the hippocampus to measure levels of Arc, mGluR5 and GluA1 mRNA (Figure 2a). The other half of the brain was used to prepare hippocampal lysate to measure protein levels of Arc (Figure 2b), mGluR5 (Figure 2c) and GluA1 (Figure 2d). The findings showed that there was upregulation of Arc mRNA and protein expression which could underlie the enhanced mGluR‐LTD seen in APP/PS1 mice. Upregulation of mGluR5 and GluA1 proteins were not mirrored by significant changes in mRNA expression, indicating that increased protein levels likely resulted from changes in protein expression or stability. As Arc protein levels are increased in APP/PS1 mice at 7 months and likely contribute to the enhanced mGluR‐LTD, we asked whether the reduction of mGluR‐LTD resulting from MK2 deletion was a consequence of decreased expression of Arc. To address this, we prepared hippocampal protein lysates from APP/PS1, WT, MK2 knockout (MK2 KO) APP/PS1XMK2^−/−^ mice at 7 months to investigate changes in Arc, mGluR5 and GluA1 protein levels (Figure 3). Western blot analysis of hippocampal lysates obtained from WT, APP/PS1, MK2 KO and APP/PS1XMK2^−/−^ mice showed that there is a significant reduction in Arc expression in the hippocampus of MK2 KO compared to WT mice (Figure 3). Furthermore, deletion of MK2 in APP/PS1 mice significantly reduced Arc protein levels. Note that the levels of Arc protein are similar in hippocampal lysate obtained from MK2 KO and APP/PS1XMK2^−/−^ mice. This result supports previous findings that the MK2 cascade regulates expression of immediate early genes, including Arc (Pintchovski et al., 2009; Ronkina et al., 2011).

### 2.3 APP/PS1 mice are unable to utilize spatial cues to perform the Barnes maze task

**FIGURE 4:**
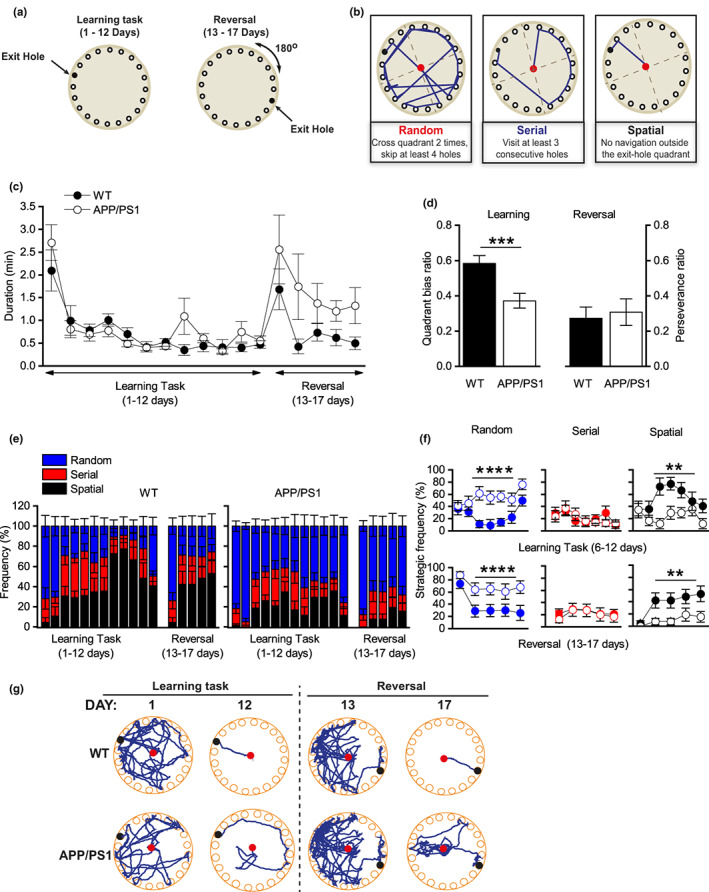
APP/PS1 mice are unable to use the spatial search strategy to perform the Barnes maze task. (a) The Barnes maze experimental setup. (b) The scoring parameters used to assess learning strategies for the Barnes maze paradigm. (c) Mean time required for WT and APP/PS1 mice to complete the task during acquisition (Days 6–12, *p* = 0.28) and reversal phases (Days 13–17, *p* = 0.124). (d) Mean quadrant bias (WT = 0.61 ± 0.042, APP = 0.37 ± 0.042; ***p = 0.001) and perseverance (WT = 0.35 ± 0.069, APP = 0.34 ± 0.076, *p* = 0.89) ratios for WT and APP/PS1 mice. (e) Percentage of time that WT (*n* = 11) and APP/PS1 (*n* = 13) mice used for random (blue), serial (red) and spatial (black) search strategies. (f) Mean frequency of strategies used in (e). During acquisition, WT mice spent more time using the spatial strategy compared to APP/PS1 mice (WT = 53.78 ± 12.87%; APP/PS1 = 24.1 ± 9.36%; ****p = 0.0009). In contrast, APP/PS1 used the random strategy at a higher frequency compared to WT mice (WT = 24.7 ± 8.3%; APP/PS1 55.21 ± 10.04%; ****p = 0.0003). During the reversal phase, WT spent more time using the spatial strategy compared to APP/PS1 mice (WT = 38.18 ± 8.64%; APP/PS1 = 10.00 ± 3.35%; **p = 0.0016) and APP/PS1 mice used the random strategy at a significantly higher frequency compared to WT mice (WT = 37.69 ± 9.09%, APP/PS1 = 69.20 ± 4.77%; **p = 0.001). (g) Trajectory taken by WT and APP/PS1 mice to complete the task.

Previous studies have shown that APP/PS1 mice are unable to efficiently learn the Barnes maze task at ages as early as 7 months (Webster et al., 2014). However, quantification of the search strategies utilized to complete the Barnes maze task have not been fully characterized in this mouse model. Previous findings have associated an inability to select the most efficient search strategy to complete the task due to dysregulation of mGluR‐LTD (Privitera et al., 2019; Wall et al., 2018). As mGluR‐LTD is enhanced in APP/PS1 mice at 7 months (Figure 1e,f), we used 7‐month‐old APP/PS1 and WT littermates to investigate the search strategy used to perform the Barnes maze task (Figure 4a,b). Mice were trained once a day for 17 consecutive days. On Day 13, the location of the exit hole was rotated 180° from its previous location, requiring the mouse to learn a new location for the exit (Figure 4a). Although both genotypes spent similar amounts of time to complete the tasks (learning and reversal phases; Figure 4c), APP/PS1 mice visited significantly less often the target quadrant as their first option during the learning phase compared to WT mice (Days 1–12; *p* = 0.001; Figure 4d). This suggests that while WT mice learn the new location of the exit hole, using the spatial cues, APP/PS1 mice may find it randomly due to an inability to select the most efficient search strategy (Figure 4b). Indeed, while WT mice replaced the use of random for spatial search strategies during training (Days 6–12), the APP/PS1 mouse failed to utilize the spatial search strategy (Figure 4e–g). During the reversal task, WT and APP/PS1 mice visited the quadrant where the exit hole was previously located (before reversal) at the same frequency (Figure 4d). However, it is interesting to note that APP/PS1 mice visited the exit hole quadrant at the same frequency during the learning and reversal phases (Figure 4d) indicating deficits in spatial learning. Corroborating this idea are findings showing that APP/PS1 mice selected a similar low proportion of spatial search strategy throughout the task (Figure 4e–g) suggesting an inability to use spatial cues. As APP/PS1XMK2^−/−^ mice showed no significant alterations in either basal synaptic transmission or plasticity compared to WT mice, we asked whether these changes reflected on their ability to utilize the spatial search strategy while performing the Barnes maze task. As indicated by similar levels of synaptic plasticity, APP/PS1XMK2^−/−^ and WT mice spent a similar amount of time performing the task (Figure S5A) and importantly the APP/PS1XMK2^−/−^ mouse increased the use of the spatial search strategy during learning the task when compared to the APP/PS1 mouse (Figure S5B–D). These findings are qualitative as the animal numbers are small. However, they suggest that the p38‐MK2 cascade mediates cognitive impairments associated with the AD pathology and is a potential pathway to be targeted to correct the cognitive decline seen early on in AD.

### 2.4 mGluR‐LTD returns to WT levels in APP/PS1 mice at 13 months

**FIGURE 5:**
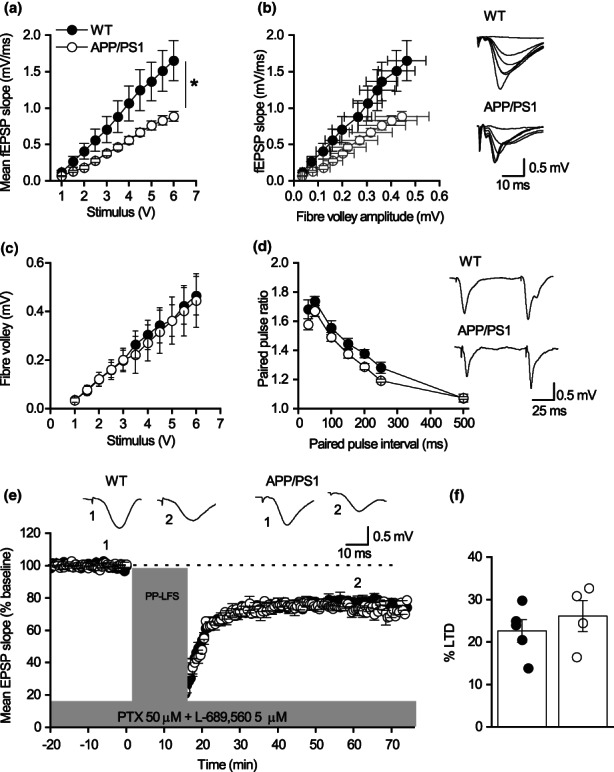
mGluR‐LTD is normalized in the hippocampus of APP/PS1 mice at 13 months of age although LTP is impaired. (a) Mean fEPSP slope against stimulus strength for WT (*n* = 5) and APP/PS1 (*n* = 8). Note the significant reduction in the fEPSP slope in APP/PS1 compared to WT; *p = 0.020. (b) Mean fEPSP slope against mean fibre volley amplitude for WT (*n* = 6) and APP/PS1 (*n* = 8) mice (*p* = 0.886). Inset: Mean fibre volley plotted against stimulus strength (1–6 V). (c) Mean fibre volley plotted against stimulus strength. (d) Mean PPR plotted against paired‐pulse interval for WT (*n* = 4) and APP/PS1 (*n* = 4). Significant difference was observed between the genotypes (*p = 0.026). Inset, traces at an interval of 50 ms from WT and APP/PS1 mice. (e) Normalized mean fEPSP slope against time for WT (*n* = 5) and APP/PS1 (*n* = 4) mice. No difference between the genotypes was observed WT: (*n* = 5, 77.4151 ± 2.65%) and APP/PS1: (*n* = 4, 73.91 ± 3.67% *p* = 0.45). Inset, traces were taken between 10–15 min (1) and LTD at 40–50 min (2) after PP‐LFS (WT: 22.58 ± 2.65% and APP/PS1: 26.09 ± 3.67%). (f) No change in LTD between 40 to 50 min after PP‐LFS was seen (*p* = 0.45; WT: 22.58 ± 2.65% and APP/PS1: 26.09 ± 3.67%).

**FIGURE 6:**
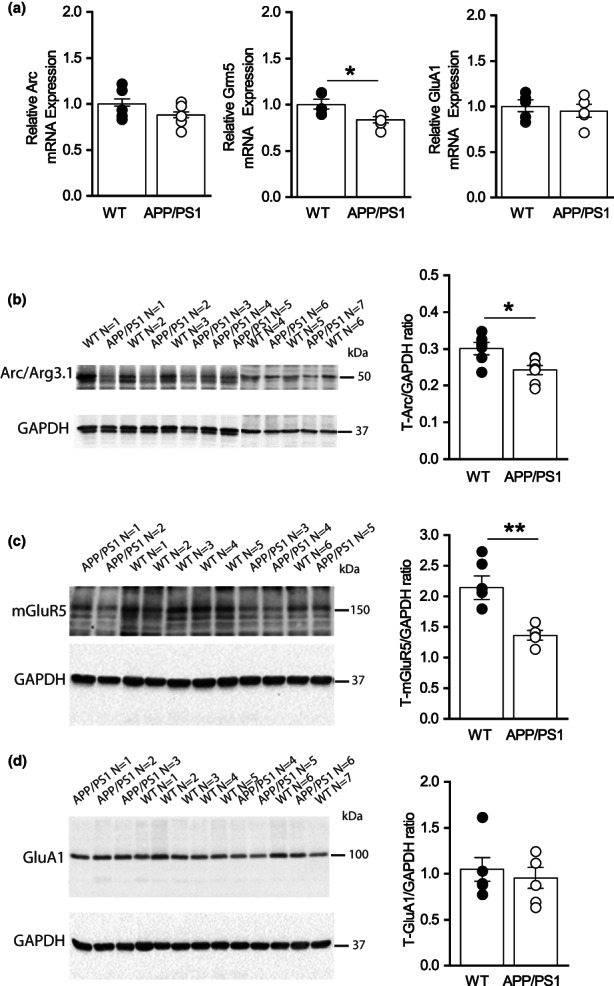
mGluR5 and Arc protein levels are reduced in the hippocampus of APP/PS1 mice at 13 months of age. (a) Bar graphs showing mRNA levels of Arc (*p* = 0.37), mGluR5 (*p = 0.02) and GluA1 (*p* = 0.53) in the hippocampus of APP/PS1 (*n* = 6) compared to WT (*n* = 5). (b–d) Blots and bar graphs showing protein expression levels of Arc (*p = 0.0133; WT: *N* = 6, APP: *N* = 7) mGluR5 (**p = 0.0041; WT: *N* = 6, APP: *N* = 5) and GluA1 (*p* = 0.577; WT: *N* = 7, APP: *N* = 6) in APP/PS1 compared to WT mice. An unpaired *t*‐test was used. *p < 0.05; **p < 0.01.

Chronic antagonism of mGluR5 in APP/PS1 mice efficiently recovered working and spatial memory at 12 months, but its efficacy was reduced with increasing age (Abd‐Elrahman et al., 2020). To investigate whether alterations in mGluR‐LTD persist as the pathology progresses, we recorded LTP and mGluR‐LTD using interleaved hippocampal slices from WT and APP/PS1 mice at 13 months. A significant impairment in the I/O curve was observed between APP/PS1 and WT littermates (*p* = 0.020; Figure 5a,b), but there was no reduction in the number of fibres stimulated (*p* = 0.886; Figure 5c). There is a reduction in paired‐pulse facilitation ratio in APP/PS1 mice (*p* = 0.026; Figure 5d), suggesting that at 13 months the impaired basal transmission is likely to result from reduced presynaptic release combined with change in postsynaptic AMPAR density. To evaluate postsynaptic changes, AMPAR‐mediated miniature excitatory postsynaptic currents (mEPSCs) were recorded from CA1 pyramidal neurons (Figure S6A–E). The amplitude of AMPAR‐mediated mEPSCs was markedly reduced in slices from APP/PS1 mice compared to WT littermates confirming reduction in the number of postsynaptic AMPARs (Figure S6B,C). There was no significant change in mEPSC intervals (Figure S6D,E) in contrast to what was observed at 7 months, suggesting that reduction in postsynaptic receptors contributes to the disruption in basal synaptic transmission observed in APP/PS1 mice at 13 months. Consistent with previous reports (Heneka et al., 2013; Ma et al., 2013), LTP was reduced in APP/PS1 compared to WT mice (*p* = 0.006; Figure S6F,G), but there was no significant difference in mGluR‐LTD (*p* = 0.452; Figure 5e,f). As mGluR‐LTD in the hippocampus of APP/PS1 returned to WT levels at 13 months, we used additional hippocampi from WT and APP/PS1 mice at 13 months to measure mGluR5, Arc and GluA1 mRNA and protein levels. No significant changes in Arc mRNA were observed between genotypes, although Arc protein levels were reduced, suggesting that the reduction of Arc is mainly regulated at the post‐transcriptional level. The expression of mGluR5 was significantly reduced at both mRNA and protein level, while no changes in GluA1 expression were present (Figure 6a–d). These findings support the hypothesis that the pathogenic contribution of enhanced mGluR‐signalling to AD is transient and manifests at early stages of the disease.

## 3 DISCUSSION

Here, we have shown that mGluR‐LTD is enhanced at CA1‐CA3 synapses of APP/PS1 mice at 7, but not at 13 months of age and that this transient enhancement of mGluR‐LTD can be prevented by reducing the MK2 signalling. Persistent Aβ production and its extracellular release has an effect on neuronal networks leading to deterioration of glutamatergic synaptic transmission and cognition (Heneka et al., 2013; Ma et al., 2013; Webster et al., 2014). As expected, deficits in LTP were present from 7 to 13 months (Figures S2 and S6). Since Aβ binds to and activates mGluR5 (Renner et al., 2010; Um et al., 2013), it is predicted that accumulation of Aβ causes persistent activation of mGluR5 signalling as the animal ages (Jankowsky et al., 2004). Consistent with this, mGluR‐LTD is enhanced in the APP/PS1 mouse at 7 months mirrored by increased expression levels of mGluR5 as well as Arc (Figures 1 and 2).

It is well‐established that Arc facilitates endocytosis of AMPA‐type GluA1 subunits underlying mGluR‐LTD (DaSilva et al., 2016; Mabb et al., 2014; Waung et al., 2008). Contrary to what was expected, we found increased levels of GluA1 protein in hippocampal lysates from APP/PS1 mice at 7 months, but not at 13 months suggesting an increase in synaptic strength at early stages of the disease. However, there was no difference in AMPAR‐mediated mEPSC amplitudes in CA1 pyramidal neurons or in the I/O relationship at basal condition between genotypes. As we used whole hippocampus to prepare protein lysates, it is possible that levels of GluA1 protein expression vary between hippocampal regions. It has been reported that reduction in GluA1 is associated with increased Arc protein expression and impaired LTP in dentate gyrus (DG) granule cells in a different mouse model of AD (Palop et al., 2007). The same study showed that LTP is intact at the CA3‐CA1 synapses between 4 and 7 months. These findings suggest that the CA3 neural network is able to maintain the optimal level of activity despite the reduced inputs from the dentate gyrus in which LTP is impaired. CA3 also receives direct inputs from the entorhinal cortex, and no information is available about its levels of activity. Nonetheless, a potential mechanism by which CA3 pyramidal neurons can respond to reduced synaptic inputs from the DG is by increasing the amount of postsynaptic GluA1 subunits. Similarly, in response to increased activity inputs from CA3, CA1 pyramidal neurons may reduce the number of postsynaptic GluA1. However, the total amount of hippocampal GluA1 is increased in APP/PS1 mice at 7 but not at 13 months suggesting increased excitability of the network at early stages of the disease.

By 13 months, mGluR‐LTD in APP/PS1 returned to WT levels (Figure 5). The mechanism(s) underlying this alteration is not clear. It could be that reduction in mGluR5 and Arc protein levels seen in APP/PS1 mice at 13 months ameliorates the mGluR5 over‐activation seen at 7 months (Figure 6). Another explanation is that a decrease in glutamate release at 13 months reduces the activation of mGluR resulting in a fall in Arc expression. Supporting this hypothesis is the decrease in glutamate release probability at 13 months (from the paired‐pulse data). We also observed a significant reduction in the I/O relationship and AMPAR‐mediated mEPSC amplitudes suggesting a reduction in AMPAR in the postsynaptic membrane. These findings define the time window where mGluR5 and Arc expression shifts from up‐ to down‐regulation in APP/PS1 mice.

The clear reduction in Arc expression between 7 and 13 months (Figures 2 and 6) suggests that mechanisms regulating Arc translation and/or degradation are dysregulated in APP/PS1 mice (Yang et al., 2016). Supporting the idea that Arc synthesis may be disrupted are findings showing that LTD induced by DHPG, a selective agonist of group 1 mGluR, is blocked at SC‐CA1 hippocampal synapses of APP/PS1 mice aged between 12 and 15 months (Yang et al., 2016). Furthermore, this study showed that suppressing the activity of PERK, a kinase which activation suppresses translation by inhibiting phosphorylation of elongation factor 2 alpha factor, corrects the inhibition of de novo protein synthesis in APP/PS1 mice and restores DHPG‐LTD to WT levels (Yang et al., 2016). Considering that two different protocols were used to induce LTD and that both protocols (synaptic versus chemical) are dependent on protein synthesis (Nosyreva & Huber, 2006), we would predict that PP‐LFS‐mediated LTD would also be impaired in APP/PS1 mice at older ages. Consistent with the hypothesis that increased Arc expression mediated the pathogenic increase in mGluR‐LTD by facilitating AMPAR endocytosis (Mabb et al., 2014; Waung et al., 2008) is the observation that deletion of MK2 in APP/PS1 mice reduced the levels of Arc protein and prevented synaptic plasticity dysfunction at 7 months (Figures 1 and 3). It remains to be elucidated whether MK2 signalling plays a direct role in regulating Arc transcription and/or translation processes (Correa & Eales, 2012; Ronkina et al., 2011). Another plausible mechanism mediating Arc accumulation is the disruption of pathways mediating Arc degradation. Although there is evidence suggesting that Arc is subject to ubiquitination and proteasomal degradation (Mabb et al., 2014; Wall et al., 2018), a recent study has suggested that Arc protein may be degraded via the endosomal‐lysosomal/autophagy pathway (Yan et al., 2018). This hypothesis is interesting as this pathway is defective in APP/PS1 mice (Abd‐Elrahman et al., 2018) and believed to contribute to the pathophysiology of AD (Menzies et al., 2017). Recent findings showed that dysfunction of the lysosomal‐autophagy system is caused by intra‐neuronal accumulation of beta cleaved c‐terminal fragment of APP in cortical neurons obtained from inducible pluripotent stem cells derived from individuals with genetic mutations in APP and PSEN1 genes (Hung & Livesey, 2018). A defective lysosomal‐autophagy system is observed in the brain of a mouse model overexpressing a human APP mutant gene and occurs before Aβ deposition is detected (Kaur et al., 2017). These findings raise the question of whether accumulation of Arc results from a combination of pathways that are defective at early stages of the disease. Thus, determining the key molecules that target Arc protein for degradation will illuminate the mechanisms triggering cognitive dysfunction in neurodegenerative disease such as AD.

Supporting our findings that alteration in mGluR‐LTD is transient are reports that chronic antagonism of mGluR5 in the APP/PS1 mouse, initiated at 6 months, reduces pathogenic features at 12, but not at 15 months (Abd‐Elrahman et al., 2020). Our findings showing that global deletion of MK2 in APP/PS1 mice prevents alterations in plasticity at 7 months (Figure 1) support a deleterious role for MK2 mediating neuronal dysfunction at a stage of the disease where changes can still be reverted. An intriguing observation from this study is the rescue of LTP in APP/PS1XMK2 KO mice. It is unclear whether Aβ‐mediated activation of mGluR5 is associated with LTP impairments seen in APP/PS1 mice. However, activation of GI‐mGluR has been shown to fine‐tune NMDAR‐mediate LTP amplitudes (Reiner & Levitz, 2018). Another important factor to consider is the effect of deleting MK2 in the brain immune cells such as microglia and astrocytes. It is well‐established that microglia and astrocytes mediate clearance of Aβ through a variety of mechanisms (Ries & Sastre, 2016). Initially, this is a beneficial process; however, as the disease progresses, these cells become over‐activated leading to increased production of pro‐inflammatory cytokines such as interleukin 1 beta (IL‐1β) and tumour necrosis factor‐α (TNF‐α) (Kaur et al., 2019). The p38‐MK2 cascade is a key regulator of synthesis and release of TNF‐α and IL‐1β in response to stressor challenges, such as exposure to Aβ (Bachstetter et al., 2011; Menon & Gaestel, 2018; Singh et al., 2017), and it is likely that global deletion of MK2 will reduce the production of these factors in APP/PS1 mice. Reduced inflammation is likely to ameliorate the pathological features observed in reactive astrocytes and activated microglia in the hippocampus of APP/PS1 mice at early stages of the disease (Ruan et al., 2009; Smit et al., 2021). As astrocytes are direct regulators of synaptic transmission and plasticity (Santello et al., 2019), it is reasonable to propose that the rescue in synaptic plasticity seen in APP/PS1XMK2 KO mice can be attributed, at least in part, to reduced activation of astrocytes mediated by disrupting the p38‐MK2 signalling. It will be interesting to use inducible genetic technology to investigate the role of MK2 activity in different cell types.

Impaired mGluR‐LTD is associated with the deficits in acquisition/consolidation of spatial learning and poor performance with task reversal (Eales et al., 2014; Mills et al., 2014; Privitera et al., 2019; Wall et al., 2018). Although spatial learning has been extensively studied in animal models of AD (Webster et al., 2014), not much information is available about their ability to utilize the spatial search strategy during learning. Our findings that APP/PS1 mice are unable to utilize the spatial cues available to replace the use of random strategy as the training progresses indicate that although the animal can complete the task, a subtle but significant cognitive decline is already happening. The observation that levels of synaptic plasticity in APP/PS1‐MK2^−/−^ mouse are normal and that they can utilize the spatial search strategy to complete the Barnes maze task (Figure S5) identifies the over‐activation of the p38‐MK2 cascade as a potential pharmacological target to correct early synaptic and cognitive impairments associated with the AD‐like pathology observed in APP/PS1 mice.

## 4 METHODS

### 4.1 Animals

Animals were treated in accordance with the Animal Welfare and Ethics Review Body Committee (AWERB), and experiments were performed under the appropriate project licenses with local and national ethical approval. All experiments involving animals have been designed in accord to the guidance provided by NC3Rs (nc3rs.org.uk). To reduce experimental variability, interleaved hippocampal slices obtained from WT and APP/PS1 littermates and APP/PS1 X MK2^−/−^ mice were used to record fEPSPs, mEPSCs. To reduce risk of bias, animal‐experimenter was blind to the animal genotype.

### 4.2 Generation of the APP/PS1XMK2 knockout mouse

In this study, C57BL/6 (wild‐type) mice as well as the APP_swe_/PS1dE9 (APP/PS1 Jackson laboratory [REF 005864] [Jankowsky et al., 2004]) and MK2 KO (Kotlyarov et al., 1999) transgenic mice were used, all of which were on C57BL/6 background. The novel APP/PS1XMK2 KO mouse was generated by cross‐breeding the APP/PS1 mouse with the MK2 KO mouse (Figure S1A–D). Protocols to genotype the APP/PS1 (http://www.informatics.jax.org/allele/MGI:3524957) and MK2 KO mice were performed by Eurofins Genomics Germany GmbH. For MK2 the following three primers were used: 5′‐CGTGGGGGTGGGGTGACATGCTGGTTGAC‐3′ (5′MK2); 5′‐GGTGTCACCTTGACATCCCGGTGAG‐3′ (3′MK2) and 5′‐TGC TCG CTC GAT GCG ATG TTT CGC‐3′ (Neomycin). Primer pair 5′MK2 and 3′MK2 amplify the wild‐type MK2 allele to yield a 500‐bp DNA fragment. Primer pair 5′MK2 and Neomycin amplify the mutated MK2 allele to produce an 800‐bp DNA fragment (Figure S1E). Genotyping of the APP/PS1 mice was carried out using the following primers: 5′‐AATAGAGAACGGCAGGAGCA‐3′ (5′PS1), 5′‐GCCATGAGGGCACTAATCAT‐3′ (3′PS1), and internal control forward 5′‐CTAGGCCACAGAATTGAAAGATCT‐3′ and reverse 5′‐GTAGGTGGAAATTCTAGCATCATCC‐3′. A fragment length of 608 bp detects the transgene (PS1dE9) and the 324 bp fragment represents an internal control present in all mice (Figure S1E). MK2 heterozygous mice were used to generate MK2 WT and MK2^−/−^(KO) mice strain as previously described (Privitera et al., 2019) (Figure S1C). Animals were kept in standard housing with littermates, and provided with food and water ad libitum and maintained on a 12:12 (light–dark) cycle. All experimental work was performed using male mice.

### 4.3 Behaviour analysis

#### 4.3.1 Barnes maze task

The behavioural cohort consisted of male APP/PS1 (*n* = 13) and WT (*n* = 11) littermates and APPswe/PS1dE9XMK2^−/−^ (*n* = 3) mice. Mice were 7‐month‐old at the beginning of the testing and were trained daily for a period of 17 consecutive days. The experimental design including the behavioural paradigm and the parameters analysed were performed as previously described (Privitera et al., 2019; Wall et al., 2018). The experimenter handled the animals daily for 1 week before training started. Before the run started, mice were kept in their original cage in a dimly lit environment for at least 30 min. To allow a preferential allocentric encoding, each mouse was then transferred to the centre of the maze covered by a 1 litre black Plexiglas beaker. Mice were left under the beaker for 30 s. During this period, the experimental light and recording system were switched on. The beaker was then removed allowing the mouse to randomly explore the maze for 10 min. The task was completed when the mouse entered the exit hole leading to the black box. If mouse failed to complete the task during the first 10 min, it was guided to the exit hole. Once into the black box the mouse was then allowed to stay into the dark enclosure for 1 min to familiarize with the environment. On Days 1–5, flavoured treats were added in the exit box as a reward for task completion. On Days 6–17, treats were awarded in the home cage on completion to prevent cued orientation of the exit box location via olfactory stimulation. To avoid olfactory cues, after each trial the arena and the exit box were cleaned twice with 70% ethanol followed by distillated water. Runs were recorded using Bird Box Camera with 700TVL Sony EFFIO CCD attached to a computer for offline analysis (Any‐MAZE v4.96, Stoelting Co.) and analysed as in (Wall et al., 2018).

### 4.4 Electrophysiology

#### 4.4.1 Hippocampal slice preparation

Hippocampal slices (400 μm) were obtained from WT, APP/PS1 and APP/PS1XMK2 KO mice. Animals were sacrificed by cervical dislocation and decapitated in accordance with the U.K. Animals (Scientific Procedures) Act (1986). The brain was rapidly removed and placed in ice‐cold high Mg^2+^, low Ca^2+^ artificial CSF (aCSF), consisting of the following (in mM): 127 NaCl, 1.9 KCl, 8 MgCl_2_, 0.5 CaCl_2_, 1.2 KH_2_PO_4_, 26 NaHCO_3_ and 10 D‐glucose (pH 7.4 when bubbled with 95% O_2_ and 5% CO_2_, 300 mOSM). Parasagittal brain slices were then prepared using a Microm HM 650 V microslicer in ice‐cold aCSF (2–4°C) and allowed to recover at 32°C for 2–6 h in aCSF with 1 mM MgCl_2_ and 2 mM CaCl_2_ before use. The CA3 region was removed in slices used for mGluR‐LTD recordings.

#### 4.4.2 Extracellular recording from hippocampal slices

Field excitatory postsynaptic potentials (fEPSPs) were recorded from interleaved slices from WT and APP/PS1 littermates and APP/PS1XMK2KO mice. An individual slice was transferred to the recording chamber, submerged in aCSF maintained at 32°C and perfused at a rate of 5 ml/min. fEPSPs stimulation and recordings details were performed as previously described in (Privitera et al., 2019). mGluR‐LTD recordings were made in the presence of 50 μM picrotoxin to block GABA_A_ receptors (Tocris) and the NMDA receptor antagonist L‐689,560 (5 μM; Tocris‐Biotechne #0742). fEPSP were evoked at 0.1 Hz with a 20 min baseline recorded at a stimulus intensity that gave 40% of the maximal response. To induce mGluR‐LTD, a paired‐pulse low frequency stimuli (PP‐LFS) consisting of 900 paired pulses with a 50 ms intervals at 1 Hz protocol was delivered at the Schaffer collaterals. For the experiments using inhibitors, after 20 min baseline, 1 μM mTEP (Tocris‐Biotechne # 2921) or 3 μM MK2 inhibitor (PF3644022‐ Tocris‐Biotechne #4279) was washed into the bath for 60 min, and another 20 min baseline was recorded before induction of PP‐LFS. LTP was induced by applying 3 X theta‐burst (10 trains of 4 stimuli of 100 Hz separated by 200 ms) repeated with a 20 s interval. For LTP and LTD analyses, fEPSP slope were normalized to the baseline. Recordings from single slices were averaged across animals, and slices were obtained from at least 3 different mice from each genotype.

#### 4.4.3 AMPA receptor‐mediated‐mEPSCs recordings

mEPSCs were recorded in CA1 pyramidal neurons from hippocampal slices obtained from WT, APP/PS1 and APP/PS1XMK2 KO mice at 7 and 13 months as described (Privitera et al., 2019). Recordings were made using glass pipettes (5–8 MΩ) filled with (mM): 135 potassium gluconate, 7 NaCl, 10 HEPES, 0.5 EGTA, 10 phosphocreatine, 2 MgATP, 0.3 NaGTP, pH 7.2, 290 mOsm. To isolate AMPA‐mediated mEPSCs, aCSF contained 1 μM TTX, 50 μM picrotoxin to block GABA_A_ receptors and 5 μM L_689,560_ to block NMDA receptors. Only one recording was made from each slice. The total number of slices were obtained from at least 3 different mice for each genotype. Recordings were performed at holding potential of −60 mV, data acquisition was performed using pClamp 10 (Molecular Devices) and analysis of mEPSCs was performed using MiniAnalysis software (SynaptoSoft) as described in (Privitera et al., 2019).

### 4.5 Protein extraction, Western blotting and quantitative PCR

Tissue homogenization and Western blotting experiments were performed exactly as described in (DaSilva et al., 2016; Eales et al., 2014; Wall et al., 2018). Membranes were probed with rabbit anti‐Arc (Synaptic Systems, 1:2500); rabbit anti‐glutamate receptor 1 (Millipore # AB1504, 1:1000), rabbit anti‐metabotropic glutamate receptor 5 (EMD Millipore # AB5675, 1:1000), rabbit anti‐beta tubulin antibody (abcam, ab6046, 1:1000) and mouse anti‐Glyceraldehyde 3‐phosphate dehydrogenase (GAPDH; abcam, ab8245, 1:5000) antibodies followed by goat anti‐Rabbit IgG‐HRP H + L (Cell Signaling, 1:10,000) and goat anti‐Mouse IgG HRP LC (Jackson ImmunoResearch, 1:20,000) secondary antibodies. Blots were imaged using the ChemiDoc™ MP Imaging System (Bio‐Rad).

#### 4.5.1 RNA extraction, cDNA synthesis and quantitative PCR

Hippocampi from WT and APP/PS1 mice were collected, submerged in RNAlater® and stored at −20°C until processed. RNA extraction and cDNA synthesis were performed as stated in (Wall et al., 2018). Primer design and qPCR reactions were performed as previously described (Lake et al., 2019). Relative quantification of *Arc, GluA1* and *Grm5* mRNA was performed using the 2^−ΔΔ*C*t^ method (Livak & Schmittgen, 2001). Data were normalized to the geometric mean of beta‐actin, GAPDH and GPI (Glucose‐6‐Phosphate Isomerase) expression and presented as expression relative to that in WT mice. Primer sequences for Arc, mGluR5, GluA1, GAPDH and β‐actin have been described in (Lake et al., 2019; Wall et al., 2018).

### 4.6 Statistical analysis

Statistical analysis was performed using Statistical Package for the Social Sciences v27 and GraphPad Prism software. LTP, LTD and input/output data were analysed using one‐way ANOVA followed by multiple comparisons tests. Behavioural data were analysed using two‐way repeated measures ANOVA. mEPSCs were compared using one‐way ANOVA and the Mann–Whitney test. Significance was set at *p* ≤ 0.05. Values represent mean ± SEM.

## AUTHOR CONTRIBUTIONS

SALC designed the project, obtained the funding, prepared figures and wrote the manuscript. SALC and LP designed behavioural paradigms. LP performed the behavioural runs and field recordings, analysed the data and prepared the figures; ELH organized the colony and genotyping, performed and analysed field recordings; LBD and ML performed and analysed Western blots; JM designed and performed the RT‐qPCR experiments and revised the manuscript; MJW performed and analysed the patch‐clamp experiments, prepared the figures and revised the manuscript.

## CONFLICT OF INTEREST

None of the authors declares any conflicts of interests.

## Supporting information


Figure S1

Figure S2

Figure S3

Figure S4

Figure S5

Figure S6

Supporting Information

## Data Availability

The data that support the findings of this study are available from the corresponding author upon reasonable request.
