# Supplementary figures and images for: The MK2 cascade mediates transient alteration in mGluR‐LTD and spatial learning in a murine model of Alzheimer's disease
Source: Aging Cell. 2022 Sep 22;21(10):e13717. doi: 10.1111/acel.13717 (PMC9577942; doi:10.1111/acel.13717)

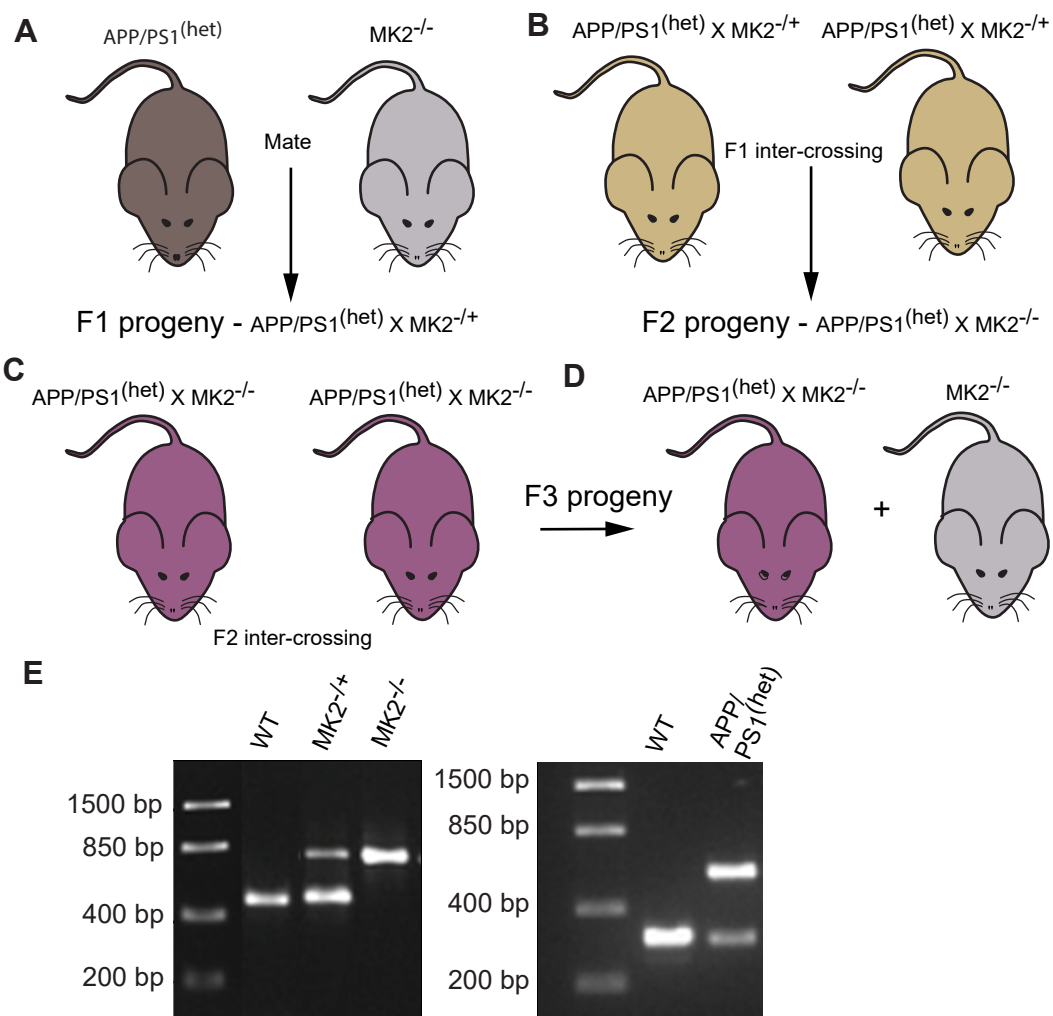

Supplement: Supplementary file 1 — Figure S1 [file ACEL-21-e13717-s002.pdf]

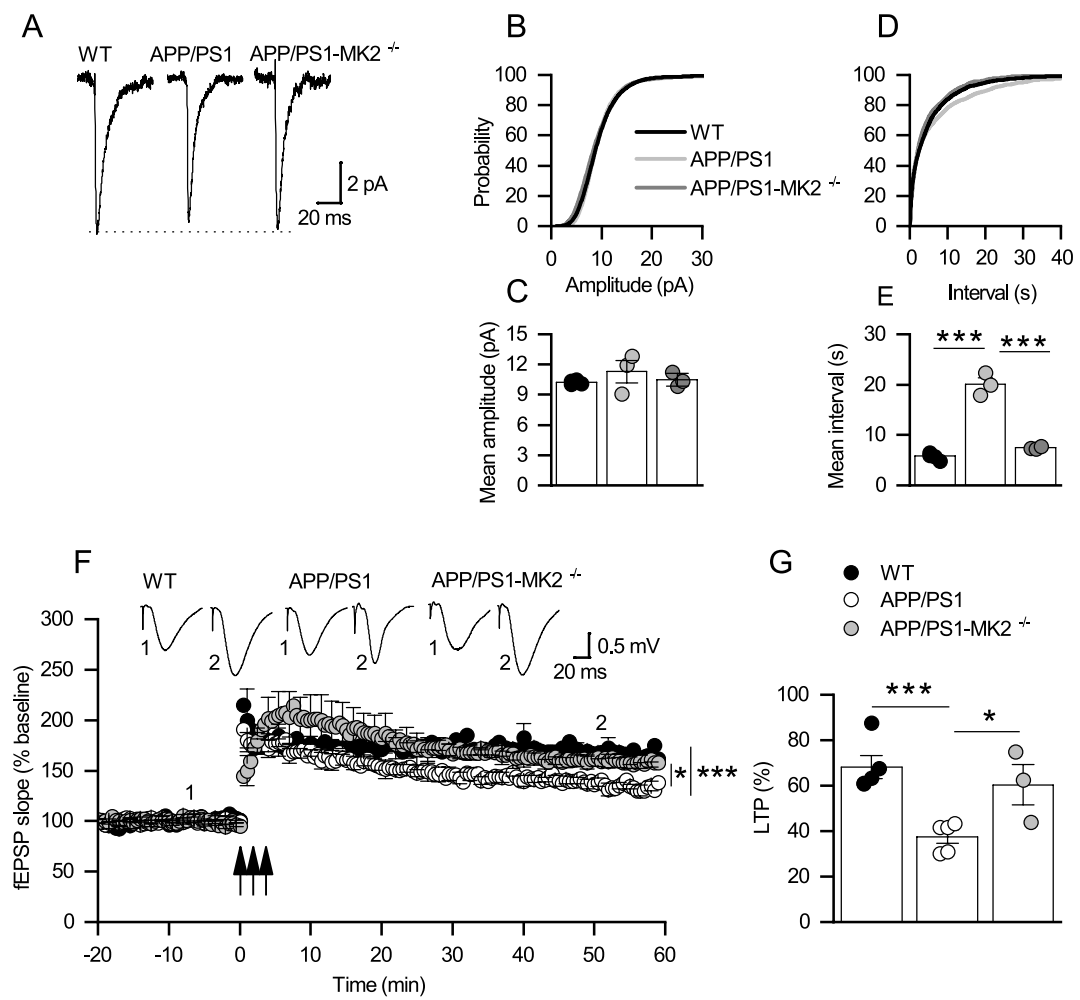

Supplement: Supplementary file 2 — Figure S2 [file ACEL-21-e13717-s001.pdf]

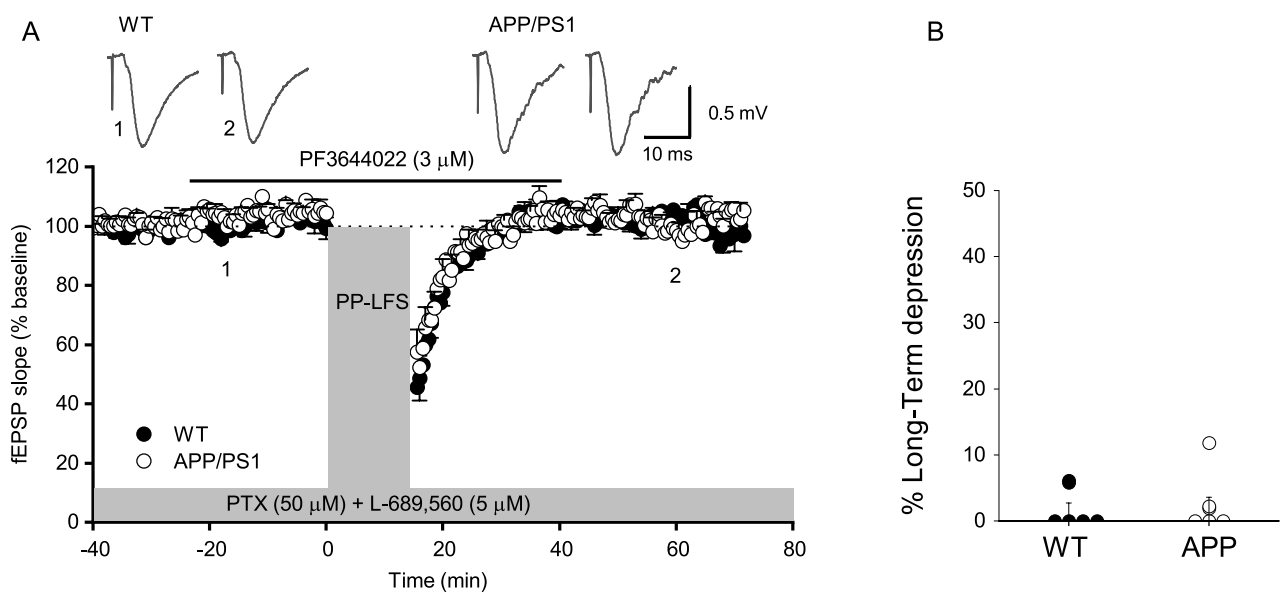

Supplement: Supplementary file 3 — Figure S3 [file ACEL-21-e13717-s006.pdf]

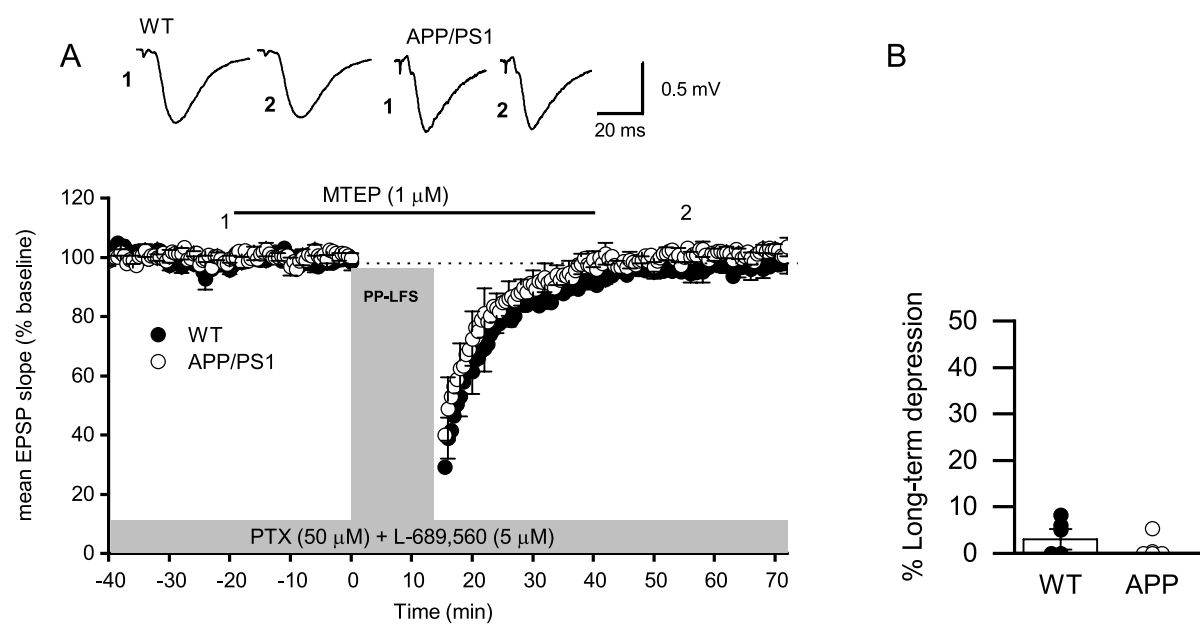

Supplement: Supplementary file 4 — Figure S4 [file ACEL-21-e13717-s003.pdf]

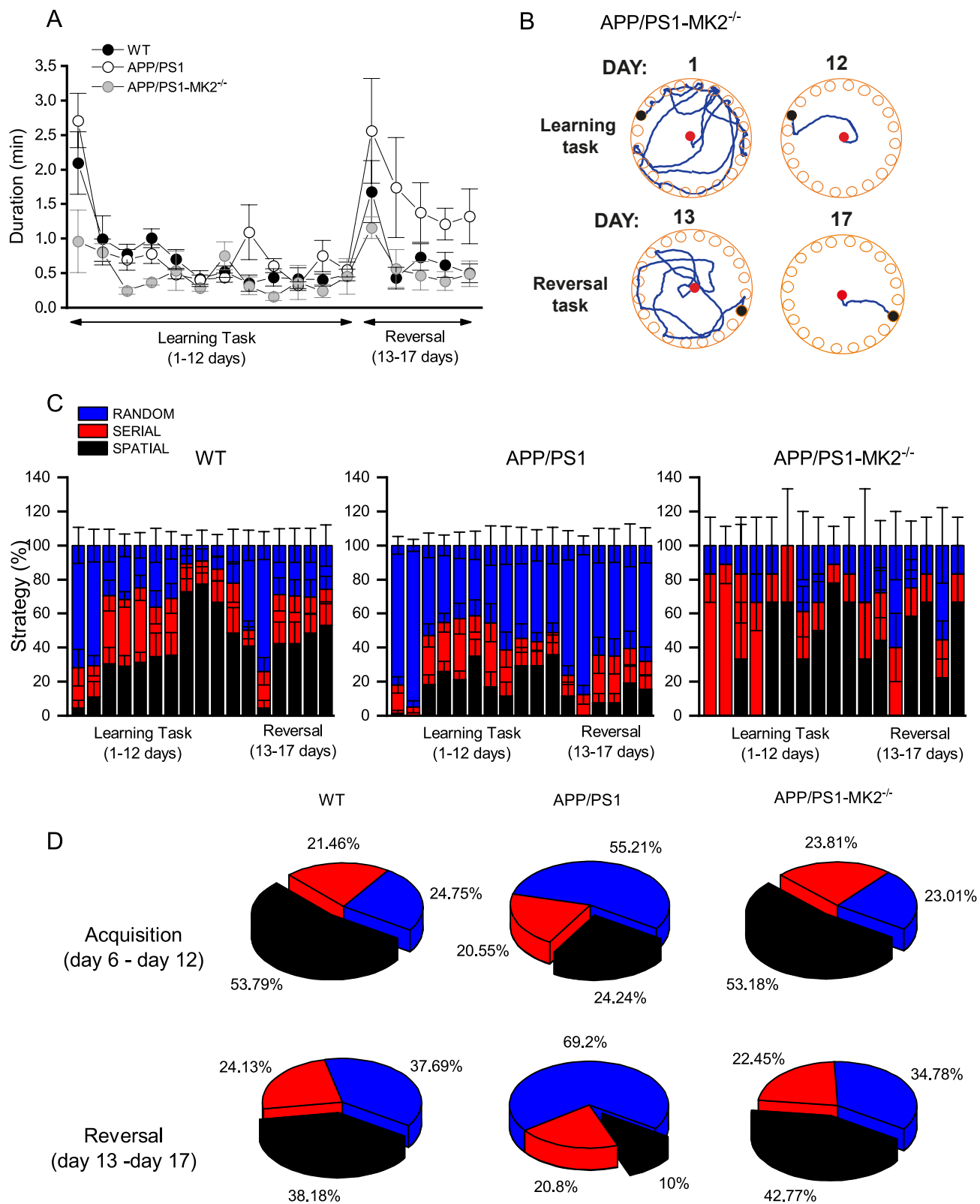

Supplement: Supplementary file 5 — Figure S5 [file ACEL-21-e13717-s004.pdf]

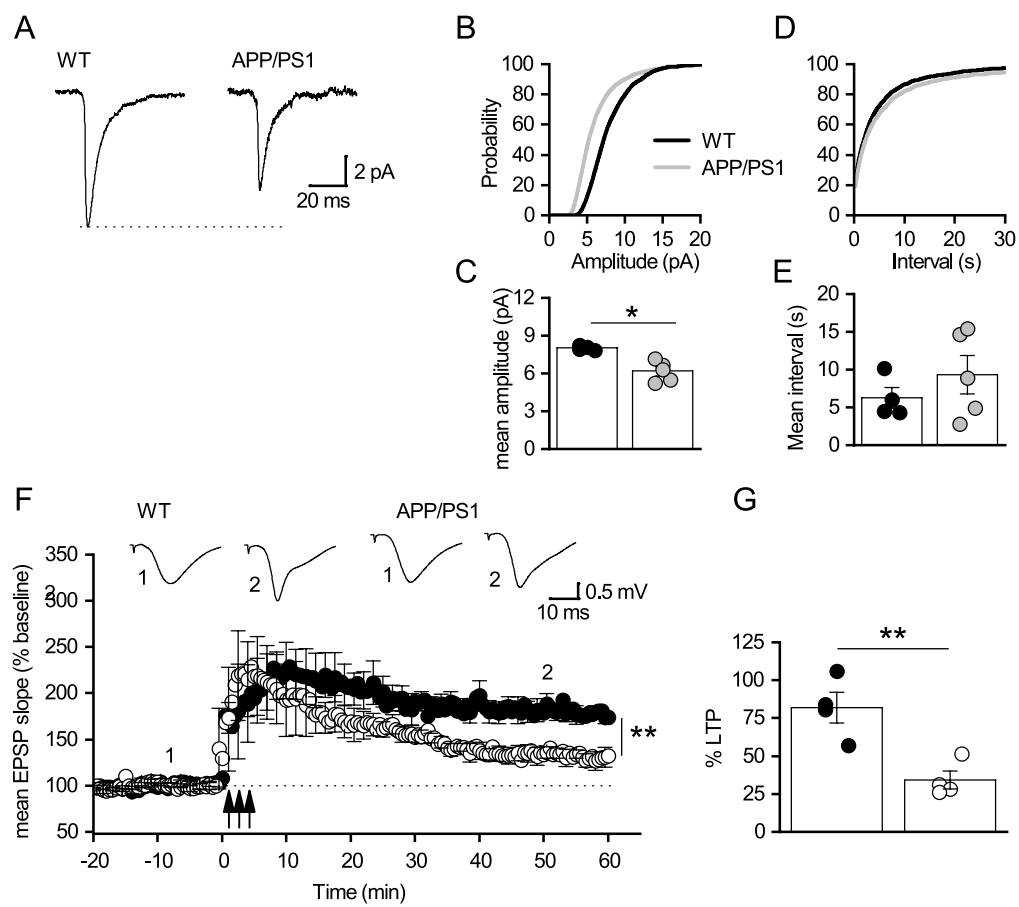

Supplement: Supplementary file 6 — Figure S6 [file ACEL-21-e13717-s007.pdf]
