# Supplementary material for: The MK2 cascade mediates transient alteration in mGluR‐LTD and spatial learning in a murine model of Alzheimer's disease
Source: Aging Cell. 2022 Sep 22;21(10):e13717. doi: 10.1111/acel.13717 (PMC9577942; doi:10.1111/acel.13717)
Supplement: Supplementary file 7 — Supporting Information [file ACEL-21-e13717-s005.docx]

Supplementary Legend Figures

Figure S1- **Generation of the novel APP/PS1 X MK2 KO mice.** **(A)** Breeding strategy to generate the APP/PS1 X MK2^-/-^ mouse. **(B)** Gels confirming genotype of the offspring using PCR assay.

Figure S2- **Deletion of MK2 in APP/PS1 mice prevents LTP impairment observed at 7 months.** **(A)** Average mEPSC waveforms from WT, APP/PS1 and APP/PS1-MK2^-/-^ mice (average of 50-70 mEPSCs aligned on rising phase). **(B)** Cumulative probability plot for mEPSC amplitudes from WT (n=14, 3m), APP/PS1 mice (n=10, 3m) and APP/PS1-MK2^-/-^ mice (n =17, 3m). (**C)** Graph plotting mean mEPSC amplitude recorded from WT (mean amplitude 10.26 ± 0.11 pA), APP/PS1 (11.3± 1.1 pA) and APP/PS1-MK2^-/-^ (10.5±0.63 pA) mice. There is no significant difference between the amplitudes across genotypes (p=0.5042). The points are mean mEPSC amplitudes from multiple recordings averaged per mouse. **(D)** Cumulative probability plot for mEPSC intervals for WT, APP/PS1 and APP/PS1-MK2^-/-^ mice. **(E)** Graph plotting mean mEPSC intervals recorded from WT (mean interval 5.79 ± 0.35 s), APP/PS1 (20.1±1.26 s) and APP/PS1-MK2^-/-^ (7.48 ± 0.16 s) mice. There is a significant difference between the genotypes (***p=1.0803X10^-5^), with the interval significantly longer in APP/PS1 mice. **(F)** Normalised mean fEPSP slope against time for WT (n=5), APP/PS1 (n=5) and APP/PS1-MK2^-/-^ (n=3) mice. Following a 20 min baseline, 3 theta burst stimulations was used to induce LTP and fEPSPs recorded for 1 hr after stimulation. Significant difference between genotypes were observed (***p=0.0033) and for APP/PS1-MK2^-/-^ compared to APP/PS1 (*p=0.047), but not difference between APP/PS1-MK2^-/-^ and WT (p>0.99, WT: 168.14± 5.02; APP/PS1: 137.60±2.90; APP/PS1-MK2^-/-^:160.41 ± 8.98). fEPSP traces were taken between 10-15 min (1) and 45-55 min (2). **(G)** Mean percentage potentiation (***p=0.0033) in fEPSP slope (LTP) between 45 to 55 min after LTP induction (WT: 68.14 ± 5.02%; APP/PS1: 37.60 ± 2.90%; APP/PS1-MK2^-/-^: 60.41 ± 8.98%). One-way ANOVA followed by Bonferroni’s test.

Figure S3- **Enhanced mGluR-LTD** **seen in APP/PS1 is mediated by MK2 activity.** (A) Normalised mean fEPSP slope plotted against time for WT (n=4) and APP/PS1 (n=5) mice. After a 20 min baseline, PF-3644022 (3 µM) was perfused into the recording chamber for a total 60 min with fEPSPs recorded for at least 50 min after PP-LFS (WT: 101.63±2.73% and APP/PS1: 102.09±3.62% p=0.523). Inset, fEPSP traces taken between 10-15 min (1) and LTD at 40-50 min after PP-LFS (2). **(B)** LTD is blocked in presence of MK2 inhibitor. fEPSP slope (LTD) normalised to the baseline (p=0.21, WT: -1.63±2.73% and APP/PS1: -2.09±3.62%).

Figure S4- **Enhanced mGluR-LTD seen in APP/PS1 mice is mediated by mGluR5.** (A) Normalised mean fEPSP slope plotted against time for WT (n=5) and APP/PS1 (n=5) mice. After 20 min baseline, mTEP (1 µM) was perfused into the recording chamber for a total of 60 min and fEPSPs recorded for at least 50 min after PP-LFS (WT: 97.01±2.20% and APP/PS1: 100.78 ±1.76% p=0.218). Inset, traces taken between 10-15 min (1) and LTD at 40-50 min after PP-LFS (2). **(B)** mGluR-LTD is blocked in presence of mGluR5 inhibitor. fEPSP slopes measured between 40 to 50 min after PP-LFS and normalised to baseline (p=0.218; WT: 2.99±2.20% and APP/PS1: 0.78±1.76%).

Figure S5- **Deletion of MK2 in APP/PS1 mice prevents their inability to utilise the spatial search strategy. (A)** Average time to complete the task (WT: n=11; APP: n=13 and APP/PS1-MK2^-/-^: n=3). **(B)** Trajectory taken by the APP/PS1-MK2^-/-^mice to complete the task. **(C)** Percentage of time that mice utilised random (red), serial (blue), and spatial (black) search strategies (n=6 for WT and APP/PS1; n=3 for APP/PS1XMK2^-/-^). **(D)** Mean frequency of strategies used in (C). Note that APP/PS1-MK2^-/-^ and WT mice utilised more the spatial strategy compared to APP/PS1. Acquisition (spatial %): WT=53.79±6.83; APP/PS1=24.24±3.79, APP/PS1-MK2^-/-^=53.18 and reversal (spatial %): WT=38.18±8.64; APP/PS1=10.0±3.15; APP/PS1-MK2^-/-^ =42.77±13.48.

Figure S6**- LTP is impaired in the hippocampus of APP/PS1 mice at 13 months.**

**A)** mEPSC waveforms from WT and APP/PS1 mice (average of 50-70 mEPSCs aligned on rising phase). **(B)** Cumulative probability plot for mEPSC amplitude from WT (n=15, 4m) and APP/PS1 mice (n=20, 5m). **(C)** Graph plotting mean mEPSC amplitudes recorded from WT (mean amplitude 8.01 ± 0.1 pA), APP/PS1 (6.18±0.36 pA) mice. The mEPSCs have a significantly smaller amplitude in APP/PS1 mice (Mann Whitney: U=20, Z=2.33, *p=0.01587). The points are mean amplitudes from individual mice. **(D)** Cumulative probability plot for mEPSC intervals for WT and APP/PS1 mice. **(E)** Graph plotting mean mEPSC intervals recorded from WT (mean interval 6.24 ± 1.36 s) and APP/PS1 (9.34 ± 2.5 s) mice. There is no significant difference between the genotypes (Mann Whitney: U=7, Z=-0.617, p=0.0556). **(F)** Normalised mean fEPSP slope against time for WT (n=4) and APP/PS1 (n=4) mice. After a 20 min baseline, LTP was induced and fEPSPs recorded for at least 1 hr after stimulation (WT: 181.60±13.86%; APP/PS1: 134.28±5.78 %). Traces taken between 10-15 min (1) and 45-55 min after LTP induction (2). **(G)** Significant reduction in the percentage of fEPSP slope (LTP) between 45 to 55 min after induction (**p=0.006) WT: 81.8 ±10.05%; APP/PS1:34.29 ± 5.78%).
